# The optimized endolysosomal degradation assay with standardized enzyme activity represents an orthogonal method for predicting protein immunogenicity and T cell epitopes

**DOI:** 10.3389/falgy.2026.1936335

**Published:** 2026-09-23

**Authors:** Elif Öztemiz, Christof Regl, Hans Brandstetter, Christian G. Huber, Kitty Verhoeckx, Gabriele Gadermaier

**Affiliations:** 1 Department of Biosciences and Medical Biology, University of Salzburg, Salzburg, Austria; 2 Center for Tumor Biology and Immunology (CTBI), University of Salzburg, Salzburg, Austria; 3 Division Internal Medicine and Dermatology, University Medical Centre Utrecht, Utrecht, Netherlands

**Keywords:** allergen, allergenicity assessment, antigen processing, cathepsin, endolysosomal, mass spectrometry, stability, T cell epitope containing peptides

## Abstract

**Introduction:**

The endolysosomal degradation assay mimics endolysosomal processing in antigen-presenting cells in vitro and can be used to evaluate protein immunogenicity and T cell epitopes. As previous endolysosomal degradation results demonstrated significant variability, we developed an optimized protocol to ensure the reproducibility and reliability of the assay.

**Methods:**

Different buffers were tested regarding their influence on the assay pH. Cleavage of ZFR-AMC to evaluate cathepsin activity was performed prior to endolysosomal degradation. Allergens from birch pollen (Bet v 1) and horse chestnut (Aes h 1) were degraded by endolysosomal proteins from the murine dendritic cell line JAWSII using an activity-standardized protocol. Long-term storage of endolysosomal proteins was evaluated, the endolysosomal half-life was calculated with a one-phase association model using densitometric gel analyses. Aes h 1 peptides were alkylated and all peptides analyzed by HPLCMS/MS. A browser-based application for visualizing the endolysosomal degradome was developed.

**Results:**

The content of the degradation and protein buffers had a significant impact on the assay pH conditions. Measurements using Z-FR-AMC showed varying proteolytic activities of different endolysosomal protein preparations. We therefore defined an endolysosomal constant of 10 μM, which was incorporated into the optimized protocol. After adjusting the endolysosomal protease amount, comparable degradation kinetics and half-lives were observed, providing consistency across different batches. Stable protease activity was observed for 63-day storage of endolysosomal proteins. Bet v 1 exhibited a half-life of one hour and multiple peptide clusters with high peptide signal intensities, some of which harbored validated T cell epitopes, were detected. In comparison, the half-life of Aes h 1 was 16 h, indicating higher proteolytic stability. Notably, alkylation of Aes h 1 endolysosomal peptides enabled superior detection of cysteine-containing fragments. Two prominent peptide clusters were identified, one harboring the region of the homologous Art v 1 T cell epitope. PeptideStudio incorporates visualization of peptide intensities and allows feature modifications to enhance the visualization of endolysomal degradomes.

**Discussion:**

The optimized endolysosomal degradation process integrates standardized protease activity and enables verified analyses of cysteine-containing peptides using PeptideStudio. This robust, orthogonal tool can support the prediction of protein immunogenicity, thereby contributing to the safety assessment of novel foods or immunogenic proteins.

## 1 Introduction

The endolysosomal degradation assay mimics endolysosomal processing of external antigens in antigen presenting cells (APCs) under *in vitro* conditions (1, 2). The assay provides information on protein stability during antigen processing and can predict potential T cell epitopes. These outcomes are valuable contributors to protein immunogenicity prediction, in particular for the assessment of novel foods, where *a priori* knowledge is desired. The method includes isolation of endolysosomal proteases from APCs and incubation with the protein of interest, thereby simulating physiological reducing and acidic conditions. The first readout of the assay is the endolysosomal half-life, defined as the time interval necessary to achieve fifty percent protein degradation. The calculated half-life links protein stability during endolysosomal degradation to immunogenicity. It has been postulated that stability in early endosomes and effective degradation in late endosomes is optimal for efficient antigen presentation (3). Therefore, proteins with moderate stability typically induce an efficient immune response. In contrast, highly stable or very unstable proteins are not processed in a timely manner for optimal antigen presentation and thus display lower immunogenicity (4, 5). The second readout is the endolysosomal degradome, which is obtained by mass spectrometric analysis of the peptides generated upon degradation with endolysosomal proteases. Endolysosomal peptides are typically displayed as clusters, which are defined as protein regions that contain several overlapping peptides. Studies with different allergens, including major allergens from birch, olive and ragweed pollen as well as peach, confirmed the location of experimentally validated T cell epitopes within endolysosomal peptide clusters (1, 2, 6–13). Therefore, the endolysosomal degradome is a complementary tool for T cell epitope analysis and prediction.

Since its first application around twenty years ago (3), the endolysosomal degradation assay has been used to investigate more than 30 allergens in the context of diagnosis and novel therapeutic options (4, 5, 14). However, it became evident that there is a need for standardization of the assay as a high diversity of half-lives was observed (1). Endolysosomal maturation is linked to acidification, as the pH gradually decreases from pH 6.8 in early endosomes to pH 4.8 in late endolysosomal compartments (15). The influence of the pH in the endolysosomal degradation assay was demonstrated using Bet v 1, which showed a ∼14-fold increased half-life when comparing pH 5.2 to pH 4.5 (4). Therefore, exact maintenance of the pH during the assay is highly relevant as it strongly influences degradation and thus half-life.

Another reason for inconsistent outcomes was attributed to the use of constant amounts of endolysosomal proteins without considering their enzymatic activity. The isolated endolysosomal fraction constitutes a protein mixture in which active endolysosomal proteases are one part among a multitude of other proteins. As a result, the composition of the fraction is not necessarily consistent between preparations, which may lead to variability in endolysosomal proteolytic activity. Therefore, using a constant protein amount across experimental replicates or studies led to varying endolysosomal half-lives. It was previously shown that endolysosomal proteins of human blood-derived DCs, murine bone marrow-derived DCs, and the murine DC cell line JAWSII demonstrated similar degradation kinetics and peptide profiles (2). They contain several members of the cathepsin family, e.g., cathepsin D, L, S, B, A, C, Z. Among these cathepsins, cathepsin S and L play a fundamental role in antigen processing (16, 17). Thus, quantities and activities of cysteine cathepsins within endolysosomal proteins strongly influence the degradation kinetics and half-life.

In this work, two allergens with different structural features were chosen as models for the optimized endolysosomal degradation assay. Bet v 1 is the major allergen from birch pollen (*Betula verrucosa*) and the most studied protein in the endolysosomal degradation assay (2, 6, 18). It has a molecular mass of 17 kDa, and a seven-stranded β-sheet which wraps around an *α*-helix incorporating a hydrophobic ligand binding cavity (19). Aes h 1 is a recently identified contact allergen of horse chestnut (*Aesculus hippocastanum*) with a mass of 5.8 kDa and a robust α/β plant defensin fold stabilized by four disulfide bonds (20).

To overcome previously observed methodological inconsistencies during endolysosomal degradation, we developed a detailed protocol for enhanced standardization to allow for assay and laboratory independent comparisons. For this purpose, we herein provided information on the pH maintenance in the assay and incorporated an *a priori* enzymatic activity measurement. In addition, endolysosomal protein storage was tested, peptide reduction and alkylation for mass spectrometry was incorporated and a browser-based peptide visualization tool was developed. Integration of those features provides a more robust and reproducible method for investigating the *in vitro* endolysosomal degradation and immunogenicity prediction of protein antigens, including previously unstudied proteins, such as novel foods, protein-based immunotherapeutics or synthetic proteins.

## 2 Materials and methods

### 2.1 Preparation of antigen presenting cells

The murine dendritic cell line JAWSII (ATCC, Virginia, USA) was cultured in minimum essential medium with *α*-modification, with nucleosides and sodium bicarbonate, without ʟ-glutamine (Sigma-Aldrich, St. Louis, MO, USA) supplemented with 15% fetal bovine serum (Catus Biotech, Tutzing, Germany), 1 mM Na-pyruvate (Sigma-Aldrich, St. Louis, MO, USA), 4 mM L-glutamine (Sigma-Aldrich, St. Louis, MO, USA), 1% penicillin/streptomycin (Sigma-Aldrich, St. Louis, MO, USA), and 5% mouse GM-CSF (Immuno Tools, Friesoythe, Germany). The JAWSII culture included adherent and non-adherent cells throughout the experiments. After reaching confluency, adherent cells were collected by a cell scraper (usage of trypsin is not recommended due to interference with the following steps) and pooled with non-adherent cells. For cultivation, JAWSII cells (2 × 10^6^) were thawed from liquid nitrogen cryostocks and cultured in a 75 cm^2^ cell culture flask with 25 mL medium. After 5 days, all harvested cells in the 75 cm^2^ flask were subcultured in a 175 cm^2^ flask with 50 mL medium. Every third day, all harvested cells were divided into two 175 cm^2^ flasks to gradually increase the culture to a final volume of 200 mL. Aliquots of 5 × 10^7^ cells were prepared in 5% DMSO medium and stored at −70 °C for subsequent endolysosomal protein isolation.

### 2.2 Isolation of endolysosomal proteins

Endolysosomal proteins from JAWSII cells were isolated by differential centrifugation. To start, 5% DMSO medium was removed from JAWSII cell aliquots (5 × 10^7^) by centrifuging at 300 g for 5 min. The cell pellet was washed with 10 mL Dulbecco's Phosphate-Buffered Saline (Sigma-Aldrich, St. Louis, MO, USA) and centrifuged at 300 g for 5 min, followed by washing with 10 mL 0.2 µm filter-sterilized fractionation buffer (10 mM Tris-acetate pH 7.0, 250 mM sucrose). The pellet was dissolved in 4 mL ice-cold fractionation buffer, and cells were lysed on ice by 25 pestle strokes using a pre-cooled glass Douncer (Kimble, Wertheim, Germany). After centrifugation at 5,525 g for 10 min at 4 °C, the supernatant was transferred to a pre-cooled 4 mL ultracentrifuge tube (Herolab, Wiesloch, Germany). To enhance the yield, the pellet containing residual intact cells was again resuspended in 4 mL fractionation buffer, lysed using the glass Douncer and centrifuged as described above. Both supernatants were centrifuged at 100,000 g for 1 hour at 4 °C. Each pellet was resuspended in 150 µL fractionation buffer, pooled, and subjected to five freeze and thaw cycles by liquid nitrogen and thawing to room temperature, respectively, to disrupt organelle membranes and obtain the endolysosomal content. Protein concentration was measured by BCA assay, according to the manufacturer's protocol (Thermo Scientific, Waltham, MA, USA). Aliquots of endolysosomal proteins were stored at −70 °C for 63 days.

### 2.3 Preparation of endolysosomal degradation buffer

Endolysosomal degradation was performed in 0.1 M citrate buffer pH 4.8, 2 mM DTT (1x degradation buffer). To maintain pH 4.8 during endolysosomal degradation, preparation of the 10x degradation buffer should strictly follow the herein proposed protocol: 1 M solutions of trisodium citrate dihydrate and 1 M citric acid (anhydrous or monohydrate) were prepared separately and mixed in a 3:2 (*v/v*) ratio. Citrate stock buffers were autoclaved, aliquoted, and stored at −20 °C. Immediately before the endolysosomal degradation, 1 M DTT was each time freshly added to 1 M citrate buffer, resulting in a concentration of 20 mM DTT. To demonstrate the effect of pH adjustment of a citrate stock buffer, another 1 M citrate buffer was titrated to pH 4.8 with NaOH, diluted up to 0.1 M; the pH was measured and compared to the citrate buffer without pH adjustment.

The influence of the protein buffer in which the protein of interest was dissolved, on the final assay pH was evaluated. Therefore, sodium phosphate buffer pH 6.0, sodium phosphate buffer pH 7.0, sodium phosphate buffer pH 7.6, and Tris buffer pH 8.6 were used at concentrations of 20, 30, 50, 75, and 100 mM, respectively, were investigated. An example pipetting scheme for two protein concentrations (1 µg/µL and 0.35 µg/µL), indicating the required buffer volumes for the endolysosomal degradation assay and the corresponding buffer simulation assay is provided in Table 1.

**Table 1:** Volumetric composition of the endolysosomal degradation assay and buffer simulation experiment.

Endolysosomal degradation assay			Buffer simulation experiment^a^		
**Components**	Based on a protein concentration of 0.35 µg/µL	Based on a protein con centration of 1 µg/µL^b^	**Components**	Based on a protein concentration of 0.35 µg/µL	Based on a protein concentration of 1 µg/µL
**Protein buffer** based on a final amount of 5 µg protein in 20 µL.	14.25 µL	5 µL	**Protein buffer** *20–100 mM, pH 6- 8.6*	14.25 µL	5 µL
**10× degradation buffer** 1 M citrate buffer, pH 4.8 at 100 mM, 20 mM DTT	2 µL	2 µL	**1 M citrate buffer** *DTT not included*	2 µL	2 µL
**Endolysosomal proteins** in fractionation buffer (10 mM Tris-acetate buffer pH 7, 250 mM sucrose)	3.75 µL (2 µg/µL endolysosomal protein concentration)	7.5 µL (1 µg/µL endolysosomal protein concentration)	**Fractionation buffer** *10 mM Tris-acetate pH 7, 250 mM sucrose*	3.75 µL (simulating 2 µg/µL endolysosomal protein concentration)	7.5 µL (simulating 1 µg/µL endolysosomal protein concentration)
**dH_2_O**	0 µL	5.5 µL	**dH_2_O**	0 µL	5.5 µL
	Total volume = 20 µL			Total volume = 20 µL	

a Volumes given are for 20 µL, final volume for pH measurements using an electrode was 5 mL.

b Typical endolysosomal degradation protocol based on previous protocols. Components are highlighted in bold

### 2.4 Cathepsin activity assay

To assess the proteolytic activity of the endolysosomal proteins, cleavage of the peptide substrate Z-FR-AMC (Bachem, Bubendorf, Germany) which emits fluorescence when its AMC fluorophore is cleaved, was measured. Despite its broad specificity, most studies refer to it as cysteine cathepsin substrate and throughout this manuscript we thus denominate Z-FR-AMC cleavage as cathepsin activity. Cathepsin activity was measured in 100 µL reaction volume using 96-well microtiter plates (Greiner, Kremsmünster, Austria). The reaction mixture consisted of 50 µL Z-FR-AMC (12.5, 25, 50, 100, 150 µM), endolysosomal proteins (1–10 µg), and 10×endolysosomal degradation buffer. 7-Amino-4-methylcoumarin (AMC) (Sigma-Aldrich, Missouri, USA) at concentrations of 2.5, 5, 10, 20, 40 µM was used for the generation of a standard curve to enable calculation of AMC cleavage of the Z-FR-AMC substrate. Endolysosomal protein was added as the final component to minimize time-dependent variation. Since AMC is light-sensitive, all steps involving this reagent were conducted under low-light conditions and reactions were kept on ice before fluorescence measurement. Fluorescence was measured at an excitation wavelength of 380 nm and an emission wavelength of 460 nm for up to 8 hours with 10-minute intervals in a pre-heated 37 °C plate reader (Tecan Infinite M200, Grödig, Austria). Fluorescence intensity values at 30 min were selected to calculate the AMC cleavage concentration based on the AMC standard curve. Raw fluorescence intensities were background-subtracted, and cathepsin activity was calculated as AMC cleavage per µg endolysosomal protein and expressed in µM/µg.

### 2.5 Optimized endolysosomal degradation assay

The endolysosomal degradation reaction included 5 µg of protein of interest, 2 µL of 10x degradation buffer, 7.5 µL of endolysosomal protein, and the final volume was adjusted to 20 µL with Milli-Q water. Within this 20 µL endolysosomal degradation mix, the maximum volume for the protein of interest was 10.5 µL, thus a minimum protein concentration of 0.48 µg/µL was required to obtain 5 µg protein. To account for varying cathepsin activities in different preparations, the amount of endolysosomal protein was calculated based on a constant of 10 µM cathepsin activity for degradation of 5 µg protein in 20 µL. The amount of corresponding endolysosomal protein (µg) was calculated as protein of interest (µg)/0.5×cathepsin activity (µM/µg). Example calculations are provided in Table 2.

**Table 2:** Examples of volumetric composition for the endolysosomal degradation assay using different cathepsin activities.

Components				
Protein of interest (µg)	5			
10×degradation buffer (µL)	2			
Calculated cathepsin activity (µM)	1	1.3	3	5
Endolysosomal protein (µg)	10	7.5	3.3	2
Fractionation buffer	Volume to reach total 7.5 µL fractionation buffer			
dH_2_O	up to 20 µL			
Total volume (µL)	20			

A master mix of the endolysosomal degradation for different time points was distributed to PCR tubes on ice to prevent enzyme activity. Samples were incubated at 37 °C in a PCR machine with top heating to prevent condensation on the lids. Endolysosomal degradation was stopped at 9 different time points (Bet v 1: 0, 0.5, 1, 2, 3, 5, 8, 16, 24 or 0, 1, 3, 5, 8, 16, 24, 48, 72 h; Aes h 1: 0, 3, 8, 16, 24, 48, 60, 72, 96 or 0, 1, 3, 5, 8, 16, 24, 48, 72 h) by denaturation at 95 °C for 5 minutes and samples were stored at −20 °C until further investigation. The 0-hour sample (non-degraded) was denatured immediately.

### 2.6 Calculation of the endolysosomal half-life

To monitor protein degradation, 10 µL of the endolysosomal degradation sample corresponding to 2.5 µg protein of interest per time point was analyzed by a reducing 15% SDS-PAGE. After staining with Coomassie Brilliant Blue R-250 (Bio-Rad, Hercules, CA, USA), protein gels were scanned with far-red light by using the Bio-Rad Chemidoc™ Touch Imaging System and analyzed by the Bio-Rad Image Lab Software. After densitometric quantification, the percentage of protein degradation at different time points was calculated by setting the time point zero as 0% degradation and was used for the calculation of the half-life. Half-life values were calculated using a one-phase association model (Y = Y0 + (Plateau-Y0) *[1-exp(-K*x)]) (21) in GraphPad Prism (version 10), with Y₀ fixed at 0 and the plateau constrained to 100 (21). When no degradation was detected initially, and onset occurred at later time points, plateau followed by a one-phase association model (Y = IF(X < X0, Y0, Y0 + (Plateau-Y0)*{1 - exp[-K*(X-X0)]}) was used, where X0 was set as the last time point before degradation was observed (22). The half-time was calculated as ln(2)/K (21), which corresponds to the endolysosomal half-life in this method.

### 2.7 Recombinant production of Bet v 1 and Aes h 1

Recombinant Bet v 1.0101 (UniProt P15494), a PR-10 protein and major birch pollen allergen and Aes h 1.0101 (UniProt Q7M1F3), an allergenic plant defensin, were purified from *Escherichia coli* as previously described (20, 23).

### 2.8 Endolysosomal degradation profile determination using mass spectrometry

Peptides generated by endolysosomal degradation were prepared for mass spectrometry by desalting with 0.2 μL C18 resin fixed pipette tips (Millipore, ZTC18M960, Germany) according to the manufacturer's protocol. To prevent disulfide bond formation in the Aes h 1 samples, peptides were reduced by tris(2-carboxyethyl)phosphine hydrochloride (TCEP) (Sigma-Aldrich, 75,259-1G, ≥ 98%, Germany) with a final concentration of 5 mM. The reaction was carried out at 55 °C for 15 minutes while shaking at 750 rpm. Alkylation was performed by adding iodoacetamide (Sigma-Aldrich, I6125-25G, ≥ 99%, USA) with a final concentration of 20 mM and incubation at RT for 10 minutes with 750 rpm shaking, protected from light.

Peptides (0.25 µg protein of interest in 1 µL) were analyzed using a Vanquish Neo UHPLC-system coupled to a Q Exactive quadrupole-Orbitrap mass spectrometer (Thermo Fisher Scientific, Germany). Peptides were separated using an Aurora Ultimate (25 cm×75 µm i.d.) C18 column (IonOpticks, Australia) at a flow-rate of 300 nL/min using eluent A [H_2_O + 0.1% (*v/v*) formic acid] and eluent B [acetonitrile + 0.1% (*v/v)* formic acid] in the following compositions: 1% B for 5.0 min, a linear gradient from 1 to 10% B in 5 min, a second linear gradient from 10%-35% B in 30 min and a third linear gradient from 35%–50% B in 10 min. This was followed by washing the column at 80% B for 5 min and re-equilibration at 1% B for 15 min. The autosampler temperature of the HPLC-system was set to 7 °C, and the column was heated to 50 °C. Peptides were ionized at 1.9 kV using electrospray ionization and analyzed in data-dependent acquisition mode using a full scan at a scan range of *m/z* 350–2,500 at a resolution setting of 70,000 at *m/z* 200, followed by five higher-energy collisional dissociation MS^2^ scans at 29% normalized collision energy using an isolation window of 2.0 *m/z* and a resolution setting of 17,500 at *m/z* 200. The automatic gain control target was set to 1e6 charges with a maximum injection time of 100 ms for the full scan and 1e5 and 100 ms for the higher-energy collisional dissociation scans. Already isolated precursor ions were dynamically excluded for fragmentation for 10 s. Data acquisition was conducted using Thermo Scientific™ Chromeleon™ 7.2 CDS.

Mass spectrometry raw files were processed in MaxQuant (v2.7.3.0) with the integrated Andromeda search engine. MS/MS spectra were searched against the amino acid sequences of Bet v 1.0101 (UniProt P15494; without N-terminal methionine) and Aes h 1.0101 (UniProt Q7M1F3; with N-terminal methionine). Because peptides were generated with a protease mixture, the digestion mode was set to “unspecific.” For Aes h 1, carbamidomethylation of cysteines was specified as a fixed modification. For both, Bet v 1 and Aes h 1, variable modifications included N-terminal acetylation, deamidation (N/Q), methionine oxidation, and formation of pyro-glutamate from N-terminal glutamine or glutamate. All identified peptides were considered irrespective of modification status. Peptide-spectrum matches were filtered at a 1% false discovery rate (FDR). To identify potential T cell epitopes and account for subsequent peptide trimming, the peptide length was restricted to a minimum of 9 amino acids (corresponding to the core binding motif) and a maximum of 30 amino acids. To monitor Aes h 1 peptides with lengths of ≥ 30, peptide length was set from 9 to 51 amino acids. Signal intensities, which correspond to semi-quantitative abundances of individual peptides, were recorded and used for estimation of peptide levels.

### 2.9 Endolysosomal degradome visualization by PeptideStudio

For improved visualization of endolysosomal degradomes, we developed a new, open-sourced peptidomics visualization tool designated as PeptideStudio. This tool was implemented in TypeScript using the React framework and bundled with Vite. It is designed as a single-page web application that is executed locally at the user-site; thus, no peptide, sequence, or intensity data is transmitted to a remote server at any stage. PeptideStudio was designed to allow upload of protein sequences in FASTA format, spreadsheet column selection, UniProt REST API retrieval, or manual entry. Peptides tables can be uploaded in XLSX, XLS, or CSV file format using the SheetJS library. Peptides can be localized to the protein sequence via user-mapped start/end columns and one or more intensity columns per e.g., timepoint.

For each intensity column, a scale was derived from either the dataset or from user-set thresholds and partitioned into user-defined number of sub-ranges (1–12) via linear interpolation. These sub-ranges are color-coded, and peptides are displayed according to peptide intensities. Peptides can be filtered by length, non-zero intensity, and optional intensity thresholds, with exclusions logged for an auditable report below the figures.

Visualizations were generated by a point-based layout engine. Overlapping peptides were assigned to non-overlapping rows via a greedy interval-packing algorithm; sequences were wrapped and paginated to a chosen physical page, and figures could be split by residue range and/or time-point. Each page was first resolved into a universal vector blueprint to generate interactive SVGs, high-resolution PNGs or JPEGs, or EPS files for vector export. Settings were saved to local storage or were exportable as JSON. The source code was released under the European Union Public License (EUPL v1.2) and is freely available at https://github.com/oztemiz-elif/peptide-studio.

## 3 Results

### 3.1 Buffer preparation and protein buffer composition strongly influence pH conditions of the assay

The endolysosomal degradation assay is performed at pH 4.8 using 0.1 M citrate buffer and reducing conditions using 2 mM DTT in the degradation buffer. To provide robust and comparable assay conditions, maintenance of the pH is critically important. Analysis of 1 M citrate buffer preparations with and without prior adjustment to pH 4.8 revealed different pH values upon dilution to up 0.1 M (Figure 1A). Notably, only 1 M citrate buffer pH 4.5 when prepared following the suggested protocol without pH adjustment showed the correct pH of 4.8 upon dilution to 0.1 M. In contrast, if the pH of the 1 M citrate buffer was adjusted to 4.8, the pH increased up to 5.1 when diluted to 0.1 M. As a higher pH typically decreases proteolytic activity in the endolysosomal degradation, preparation of the non pH-adjusted 1 M citrate buffer should exactly follow the provided protocol in the Methods section.

**Figure 1:**
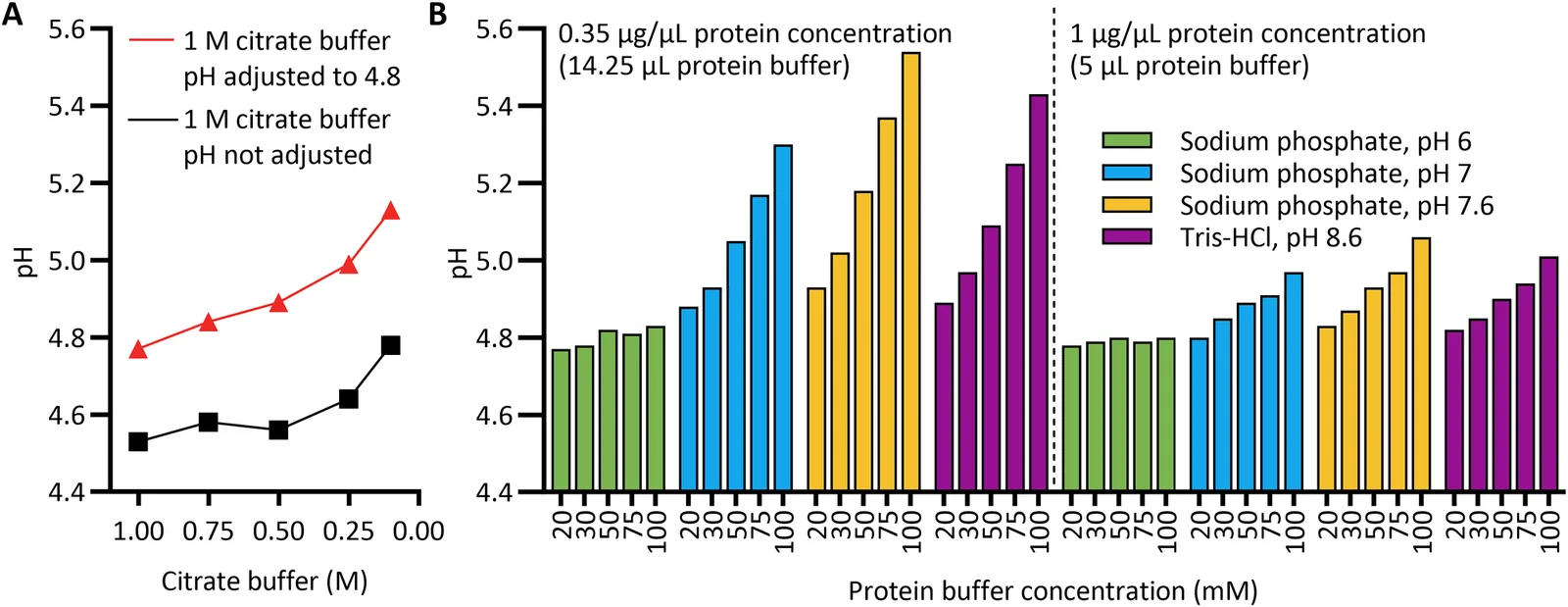
Effect of citrate buffer and protein concentration on the pH. **(A)** 1 M citrate buffer preparations were diluted up to 0.1 M to test their effect on the pH. **(B)** Endolysosomal degradation was simulated using different protein buffers to test their influence on the pH. Buffer components were mixed according to Table 1.

To simulate the influence of the protein buffer in which the protein of interest was dissolved, sodium phosphate and Tris-HCl buffers with pH ranging from pH 6–8.6 and buffer concentrations ranging from 20 to 100 mM were analyzed. The simulations were upscaled based on protein buffer volumes (14.25 and 5 µL respectively) required for samples with theoretical protein concentrations of 0.35 and 1 µg/µL (Table 1 and Figure 1B). Sodium phosphate pH 6 maintained the required pH 4.8 at all tested buffer concentrations and both simulated protein concentrations. 20 and 30 mM sodium phosphate buffers pH 7 also maintained pH 4.8 at both protein concentrations, but 50 mM and 75 mM protein buffers only at 1 µg/µL simulated protein concentration. Usage of the 20 mM phosphate pH 7.6 and 20 mM Tris-HCl pH 8.6 maintained pH 4.8 at both protein concentrations. When using higher molarities of the buffers (30 and 50 mM), the correct buffer pH could only be maintained with the 1 µg/µL sample simulation. Overall, buffers at pH 6 were robust across all conditions tested and thus would not influence endolysosomal degradation. Buffers at pH ≥ 7 preserved the desired pH 4.8 at 20 mM for both tested protein concentrations, while buffers with higher ionic strength (≥30 mM) could only maintain the pH at the higher protein concentration.

### 3.2 Cathepsin activity determination enables activity adjustment of endolysosomal proteins

To establish the cathepsin activity of the endolysosomal proteins, different Z-FR-AMC concentrations (12.5 to 150 µM) were tested (Supplementary Figure S1). The highest Z-FR-AMC concentrations (150 and 100 µM) resulted in an oversaturated fluorescence signal within 1 hour. Although 12.5 µM Z-FR-AMC showed acceptable results, the low signal intensity was considered a risk for inaccurate measurements at early time points. Both 25 µM and 50 µM Z-FR-AMC showed robust measurements with signal plateaus reached after 1.5 and 2 hours, respectively. As both concentrations were equally suitable, 25 µM Z-FR-AMC was selected as optimal concentration. The optimal amount of endolysosomal protein was 2–5 µg and was used for all subsequent analyses (Table 3).

**Table 3:** Volumetric composition of the cathepsin activity assay.

	Volume (µL)
Z-FR-AMC (50 µM) *for final 25* µ*M concentration*	50
10x degradation buffer	10
Endolysosomal protein (1 µg/µL)	2–5
Fractionation buffer	Fractionation buffer up to 5 µL if <5 µL endolysosomal protein was used
dH_2_O	35
Total volume	**100**

To calculate the cathepsin activity, fluorescent intensity values after 30 min were selected (Figure 2A), based on low standard deviations across different endolysosomal protein batches. For assay reproducibility, measured fluorescence intensities were converted into quantitative values by using a standard curve plotted by an AMC reference standard (Figure 2B). The cathepsin activity is calculated by normalizing the cleaved AMC concentration by the amount of endolysosomal protein used and expressed as µM AMC cleavage per µg of endolysosomal protein.

**Figure 2:**
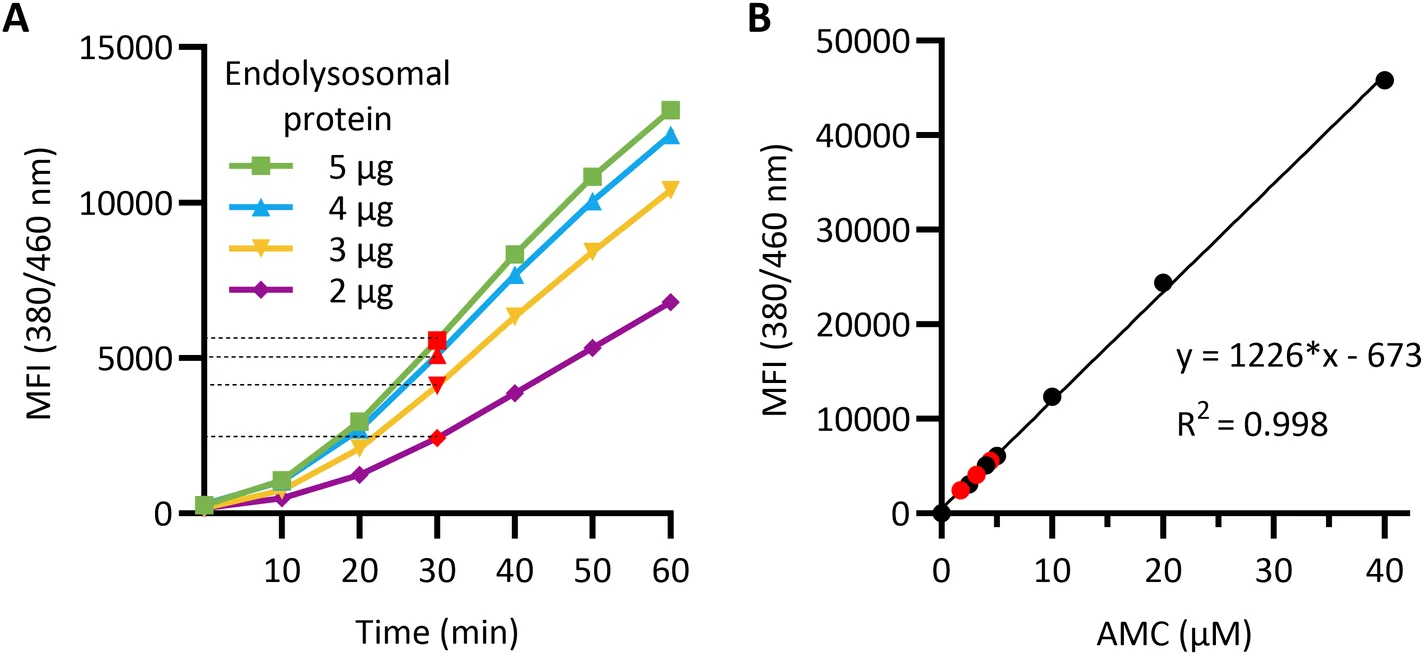
Cathepsin activity measurement and conversion of fluorescence measurement to AMC cleavage. **(A)** 25 µM Z-FR-AMC was cleaved by 2-5 µg of endolysosomal protein under endolysosomal degradation assay conditions. The first hour of the 8-hour measurement is represented, with fluorescence recorded at 10-minute intervals. **(B)** Standard curve of AMC reference in a serial dilution of 2.5 to 40 µM using measurements at 30 min was fitted by simple linear regression. The corresponding regression equation was used for calculation of cathepsin activity by converting fluorescence measured by Z-FR-AMC cleavage at 30 min (red data points in graph A) to concentration (µM) of AMC cleavage (red dots on graph B).

### 3.3 Endolysosomal constant application enhances the assay protocol

Endolysosomal activity refers to the final cathepsin activity in the endolysosomal degradation sample and is calculated by multiplying the measured cathepsin activity with the amount of endolysosomal protein used. To test the effect of endolysosomal activity on protein degradation and half-life, lower and higher amounts compared to the previous standard usage of 7.5 µg endolysosomal proteins were tested for degradation of Bet v 1. Increased endolysosomal protein amounts and thus endolysosomal activity led to an accelerated protein degradation as observed in gel electrophoresis, while lower amounts showed the opposite (Figure 3A). Consequently, the calculated half-lives were affected depending on the amount of endolysosomal proteins used.

**Figure 3:**
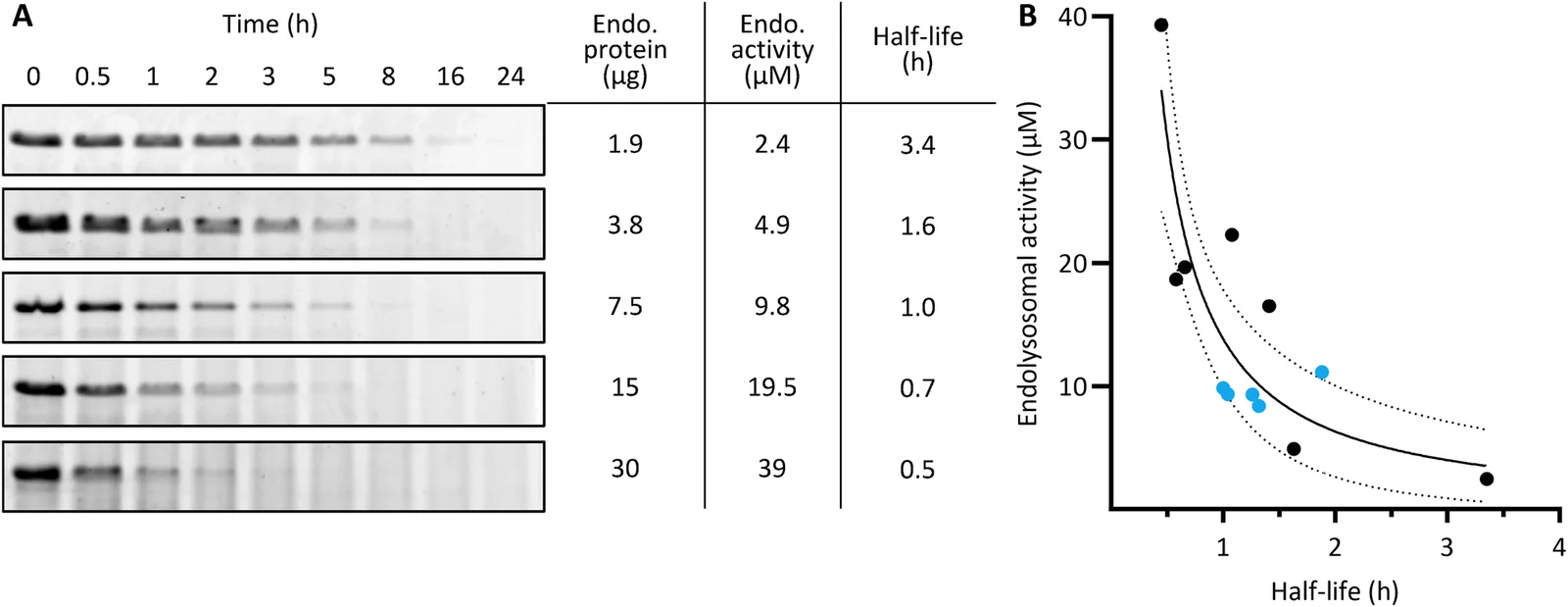
The effect of different endolysosomal activity on Bet v 1 endolysosomal halF–life. **(A)** Endolysosomal degradation of Bet v 1 analyzed by Coomassie stained protein gels. Different amounts of endolysosomal proteins (1.3 µM cathepsin activity) were used to reveal the endolysosomal activity ranging from 2.4–39 µM. Endolysosomal half-lives were obtained from densitometric analyses of protein bands. **(B)** Overall distribution of Bet v 1 half-lives according to endolysosomal degradation activity (*n* = 12). Graph generated from individual Bet v 1 endolysosomal degradation experiments. Blue points mark the nominal half-lives according to our repeated experiments. Endo. abbreviation refers to endolysosomal.

For an enhanced endolysosomal degradation assay protocol, an endolysosomal constant value was defined. This constant was selected upon analyzing endolysosomal half-lives of Bet v 1 obtained from twelve independent experiments using varying endolysosomal activities (Figure 3B). Summarized data showed that a mean endolysosomal activity of 10 µM (range 8.40–11.14 µM) was optimal for degradation of 5 µg protein, as this revealed consistent results within our repeated Bet v 1 half-life measurements (1.3 ± 0.4 hours). Therefore, 10 µM was defined as the constant value for endolysosomal activity to degrade 5 µg protein and was used in all subsequent experiments.

### 3.4 Integration of the endolysosomal constant enables standardization of the endolysosomal activity

Endolysosomal degradation of Bet v 1 and Aes h 1 were investigated using endolysosomal protein batches with different cathepsin activities. Normalization of the endolysosomal activity to 10 µM revealed a consistent half-life of 1 hour ± 0.1 SEM for seven Bet v 1 measurements (Figure 4, Supplementary Figure S2). In contrast, following the previous protocol with a fixed amount of 7.5 µg endolysosomal protein with higher cathepsin activity (2.7 µM AMC/µg), degradation was faster resulting in a lower half-life of 0.6 hours (Figure 4). Similarly, endolysosomal degradation of Aes h 1 was tested using the optimized protocol with constant 10 µM endolysosomal activity, showing a mean half-life of 16 hours ± 1.6 SEM, (*n* = 4) (Figure 4, Supplementary Figure S2). Following the previous protocol without adjusting the endolysosomal activity, Aes h 1 half-life was skewed to an earlier time point, i.e., 12 hours (Figure 4).

**Figure 4:**
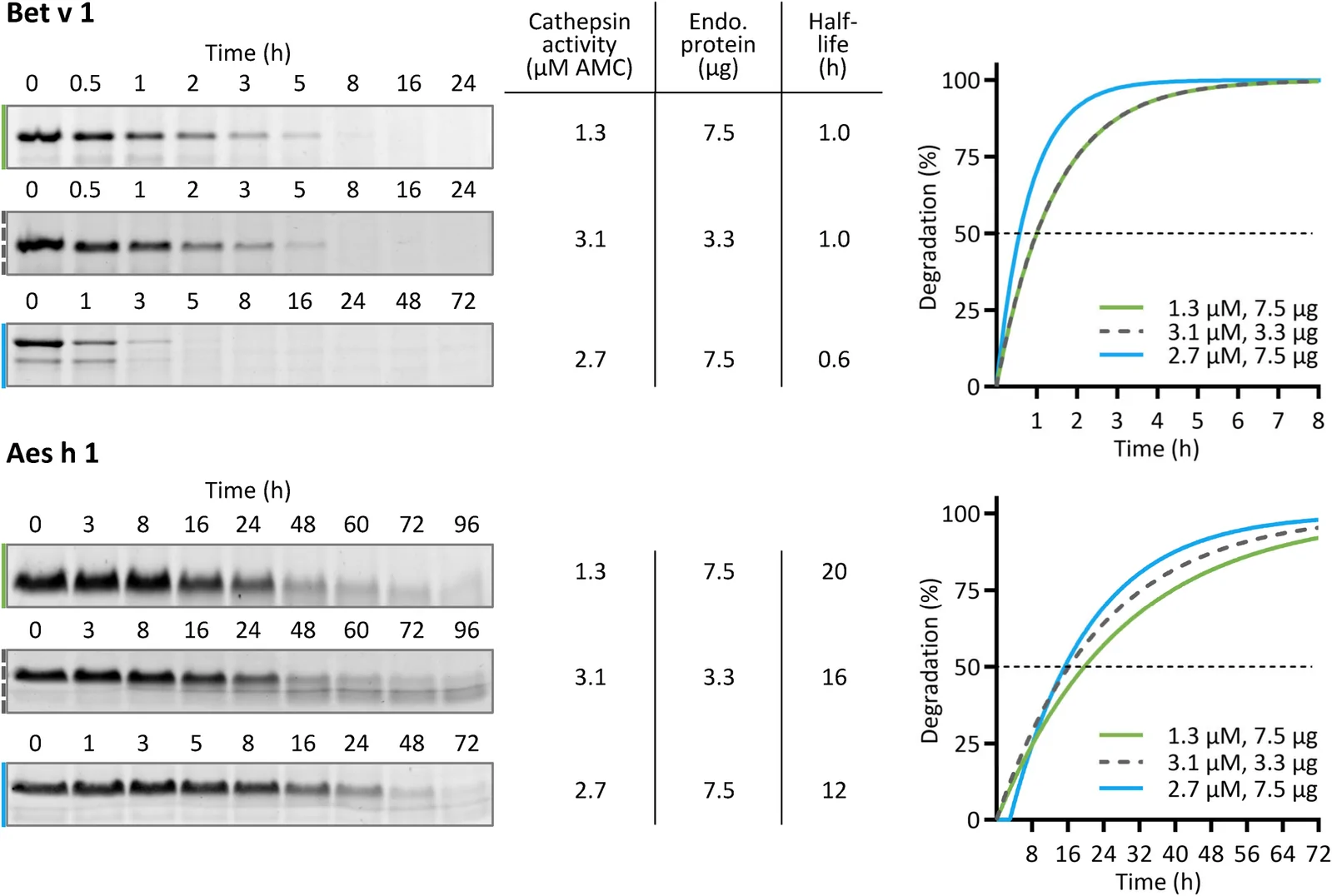
Comparing adjusted and non-adjusted endolysosomal activity for degradation of Bet v 1 and Aes h 1. Bet v 1 and Aes h 1 were degraded by endolysosomal proteins with 1.3 µM, 2.7 µM and 3.1 µM cathepsin activity. Endolysosomal protein batch with 3.1 µM cathepsin activity was adjusted to 10 µM endolysosomal activity and batch with 2.7 µM cathepsin activity was non-adjusted (7.5 µg). Half-lives were calculated from densitometric analyses of protein bands from representative gel images and degradation percentages were fitted by one-phase association. Experimental repeats of half-life calculations are shown in Supplementary Figure S2. Endo; Endolysosomal.

### 3.5 Endolysosomal protein activity remains stable at least 63 days of storage

To investigate the influence of storage at −70 °C on endolysosomal protein stability, we measured cathepsin activity of endolysosomal proteins stored up to 63 days. Cathepsin activity remained comparable to that of freshly prepared endolysosomal proteins (day 0) across three independent endolysosomal protein batches (Figure 5A). Likewise, similar degradation kinetics of Bet v 1 and Aes h 1 were observed in protein gels using an endolysosomal protein batch that was either used immediately (day 0) or after storage for 20 or 63 days. This also resulted in comparable half-lives (Figure 5B) and degradomes with identical cluster regions and similar peptide counts for both proteins (data not shown). These findings indicate that endolysosomal proteins can be stored at −70 °C for up to 63 days without compromising assay performance.

**Figure 5:**
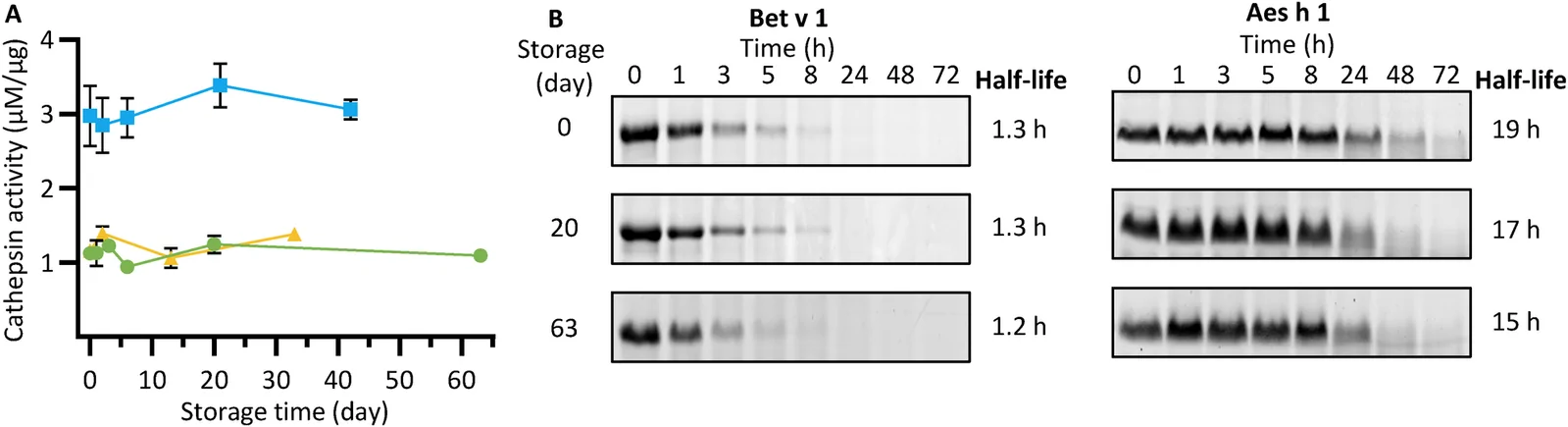
Stability of endolysosomal activity after storage. **(A)** Three independent batches (depicted as blue, green, yellow lines) of endolysosomal proteins were used to determine the effect of storage at −70 °C on cathepsin activity. **(B)** Endolysosomal degradation of Bet v 1 and Aes h 1 using the green endolysosomal protein batch **(A)**, either used directly after preparation or after storage for 20 or 63 days. Half-lives were calculated from densitometric analysis of protein bands by one-phase association equation.

### 3.6 Endolysosomal degradomes featuring peptide intensities visualized by PeptideStudio

Requirements for an improved visualization of the endolysosomal degradomes resulted in the development of an open-source browser-based tool, which was designated PeptideStudio. This tool allows to align endolysosomal peptides to the protein sequence by displaying each time point on a separate track and to incorporate mass spectrometry-derived peptide intensities using a color code (Figures 6–8).

**Figure 6:**
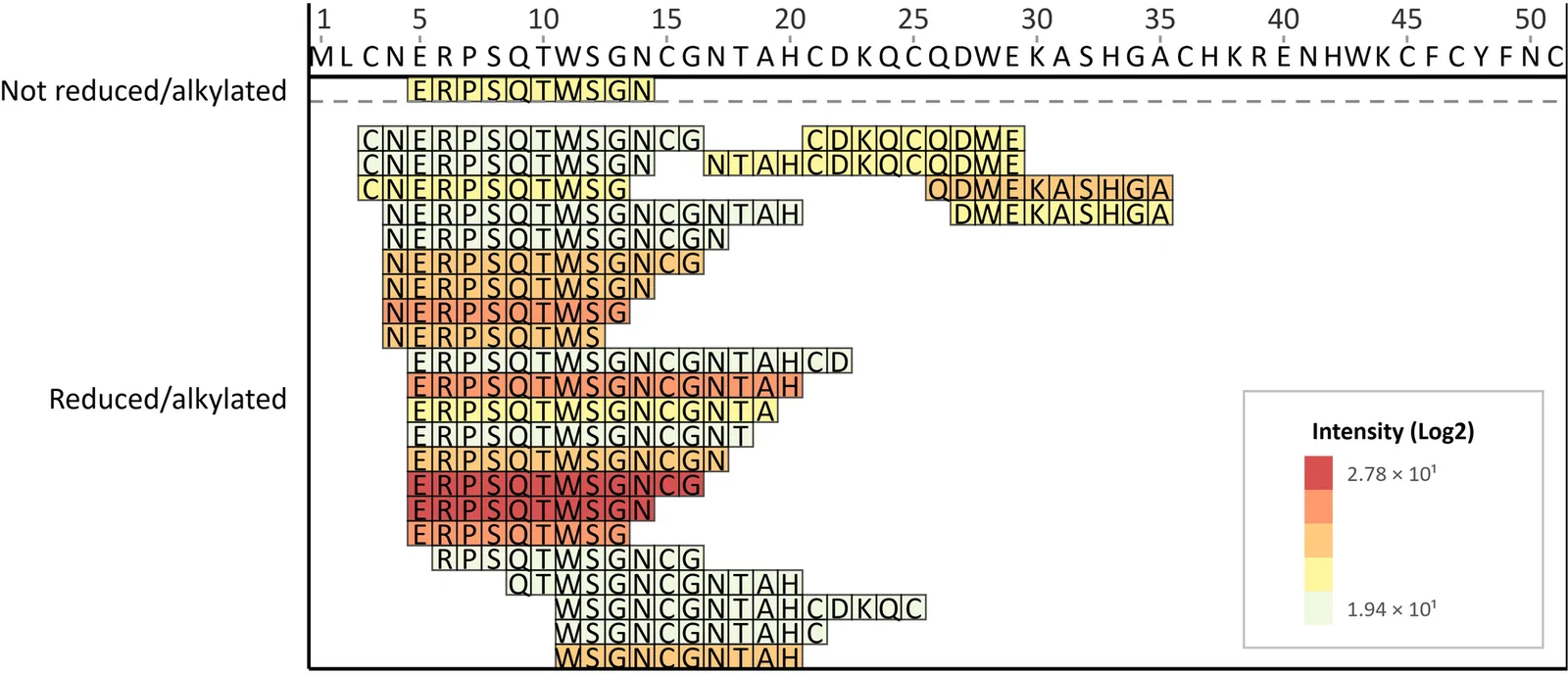
Identification of Aes h 1 peptides from non-treated or reduced and alkylated samples after endolysosomal degradation. Endolysosomal peptides were generated using the optimized endolysosomal degradation protocol (24-hour degradation). The endolysosomal peptide sample was split in half and either non-treated or reduced and alkylated prior to mass spectrometry.

**Figure 7:**
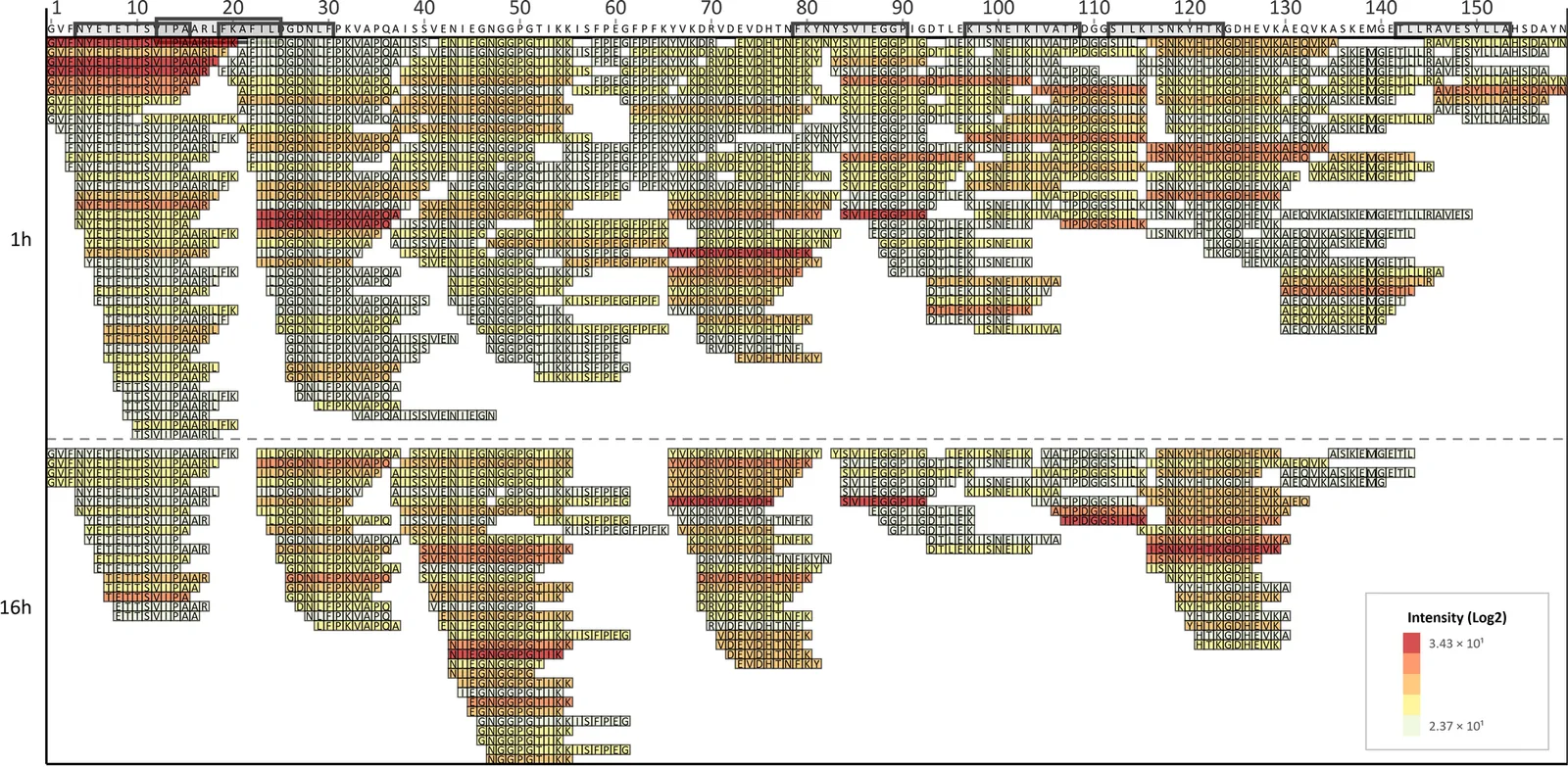
Endolysosomal degradome of Bet v 1. Two representative timepoints (1 and 16 hours) were selected. Peptides above the 25% percentile of all intensity values were included, and the peptide length was limited to 9 to 20 amino acids. The full profile is shown in Supplementary Figure S3. Experimentally validated T cell epitopes were obtained from (41) and are shown as grey boxes in the Bet v 1 sequence on top.

**Figure 8:**
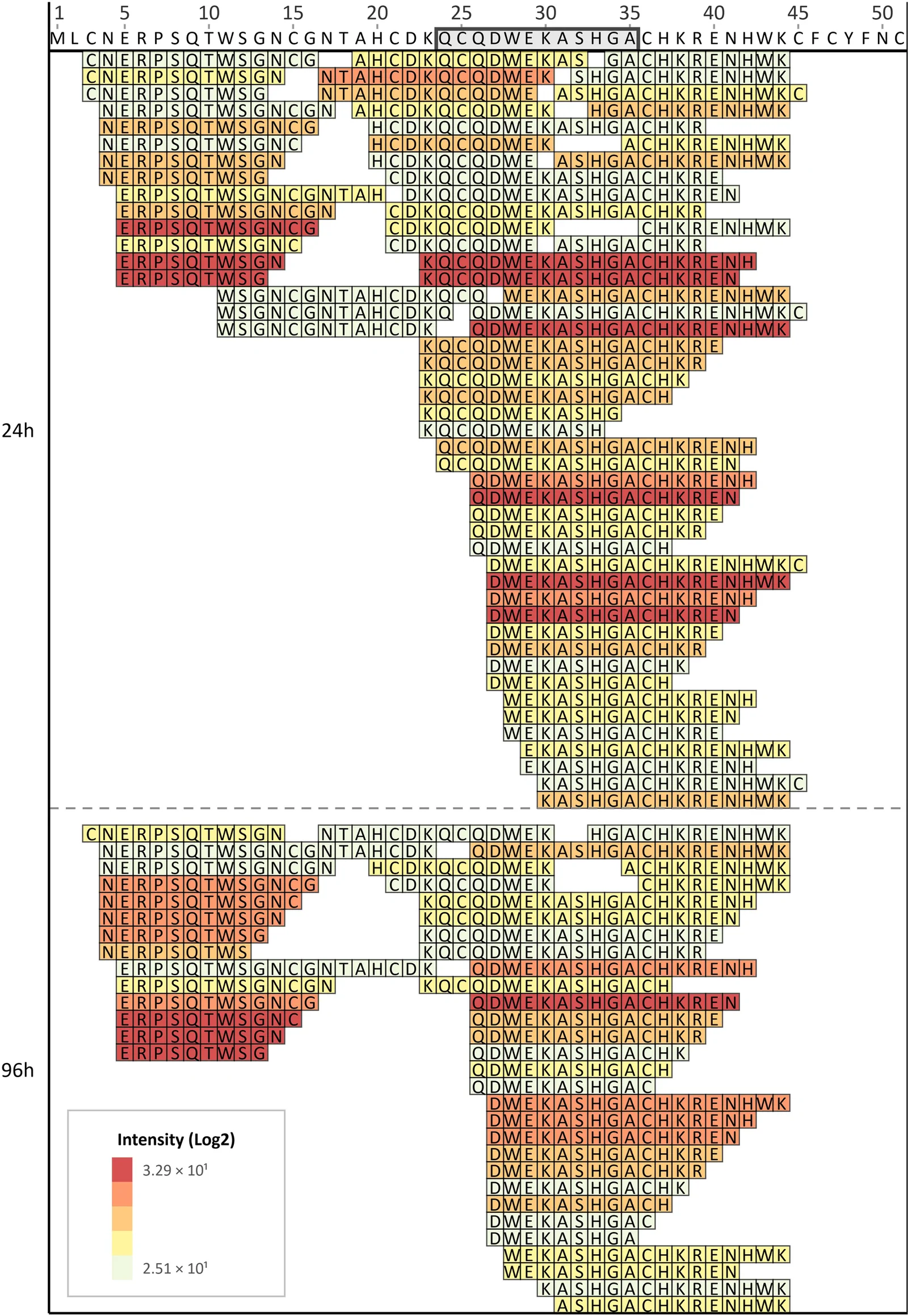
Aes h 1 endolysosomal degradome. Two representative timepoints (24-hour and 96-hour) were selected. Peptides above the 25% percentile of all intensity values were included and the peptide length was limited to 9 to 20 amino acids. The full profile is shown in Supplementary Figure S4. The immunodominant T cell epitope of Art v 1, Art v 1_25−36_ which corresponds to Aes h 1_24−35,_ is shown as grey box in the Aes h 1 sequence on top.

Ranges for peptide length and intensity can be selected according to maximum and minimum input data or selected by the user. The number and individual colors can be user edited. Intensity values can be depicted as raw intensity values or log2/log10-transformed data, and displayed in superscript, exponential, integer, or float notation. Protein regions of interest, epitopes, and/or modified amino acids can be highlighted directly within the sequence with boxes or colored lines. PeptideStudio was developed to provide an adjustable visualization of endolysosomal degradomes.

Furthermore, the tool was improved by customizable multi-tier settings. For large datasets, the protein sequence can be split into stacked vertical ranges and/or the time points distributed across multiple horizontal figures. The number of residues per row can be fixed manually or computed automatically to fill the printable page width at the configured font size. All display settings including fonts, colors, and page layout, were designed as adjustable and can be saved to and reloaded from a configuration file. These features allow preparation of figures suitable for publication by solely using PeptideStudio.

### 3.7 Reduction and alkylation ensures identification of cysteine containing endolysosomal peptides

To test the potential influence of non-desired, random disulfide bond formation of peptides after endolysosomal degradation, peptides of Aes h 1 were analyzed directly (non-treated) and after a reduction and alkylation step (Figure 6). In the non-treated sample, only one peptide was identified by mass spectrometry which did not harbor a cysteine residue. Notably, reduction and alkylation of the Aes h 1 peptides enabled identification of 28 peptides and many of the peptides contained cysteine residues.

### 3.8 Bet v 1 endolysosomal degradome presented multiple peptide clusters including high intensity peptides

The endolysosomal degradome of Bet v 1 revealed ten clusters spanning the entire protein sequence (Figure 7, Supplementary Figure S3). For the first time, peptide intensity was incorporated in the visualization, which is valuable to determine the strength of peptide clusters as well as the most prominent peptide(s) within a cluster. Experimentally validated Bet v 1 T cell epitopes were localized within the dominant peptide clusters, Bet v 1_1−22_, Bet v 1_20−36_, Bet v 181-118, Bet v 1_141−159_. Interestingly, the peptide cluster containing the immunodominant T cell epitope Bet v 1_142−156_, showed less peptides compared to the others, however, peptides were generated early during degradation and persisted until 5 hours (Supplementary Figure S3). Bet v 1 peptides containing experimentally validated T cell epitopes revealed the highest presence at 1 hour, their intensities maintained up to 3 hours of degradation and then they gradually decreased (Figure 9). The refinement of the Bet v 1 degradome, achieved through increased peptide numbers and the implementation of peptide intensities displayed in PeptideStudio, provides a clearer view of the proteolytic cleavage sites during endolysosomal processing.

**Figure 9:**
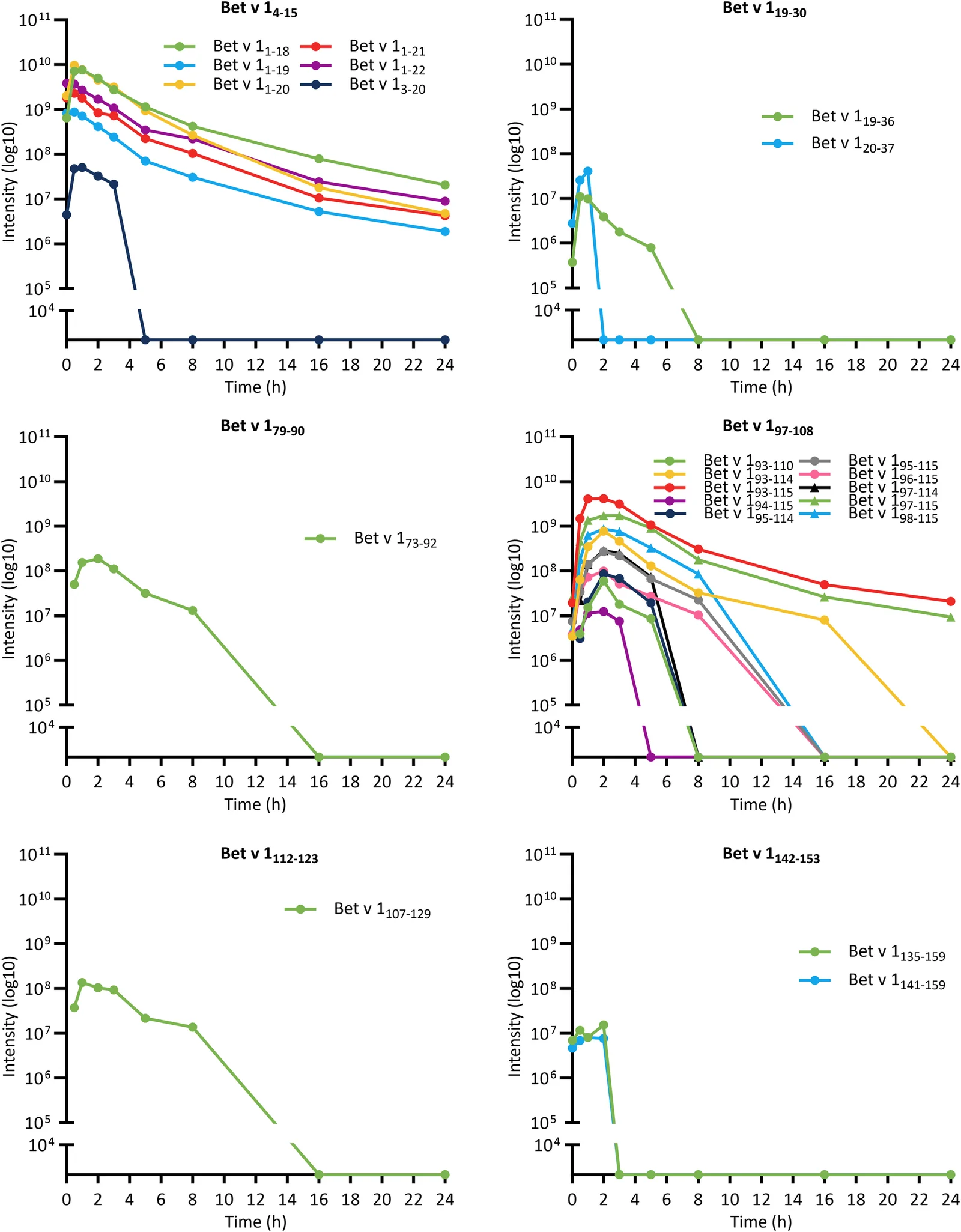
Degradation kinetics of peptides harboring Bet v 1-T cell epitope. Intensity values of peptides containing experimentally validated Bet v 1 T cell epitopes were monitored for 24 hours. T cell epitope-containing peptides were selected based on the following criteria: **(i)** length between 18 and 26 amino acids; (ii) continuous longitudinal presence with detection at minimum 3 time points; and (iii) ± 7 amino acids as flanking N- and C- terminal residues.

### 3.9 Aes h 1 degradome shows two peptide clusters encompassing the Art v 1-homologous T cell epitope

The endolysosomal degradome of Aes h 1 revealed two prominent clusters at Aes h 1_1−15_ and Aes h 1_17−45_. Interestingly, the homologous region of the immunodominant T cell epitope of Art v 1 (61% sequence identity) is within the strong cluster Aes h 1_17−45_ (Figure 8). The endolysosomal degradome of Aes h 1 showed an increase in peptide count up to 48 hours, followed by a decline in both the number of peptides and the total peptide intensity values (Supplementary Figure S4). Degradation kinetics of Aes h 1 peptides corresponding to the immunodominant T cell epitope Art v 1_25−36_, revealed the highest presence of these peptides at 24 hours which was maintained up to 48 hours and started to decrease gradually (Figure 10).

**Figure 10:**
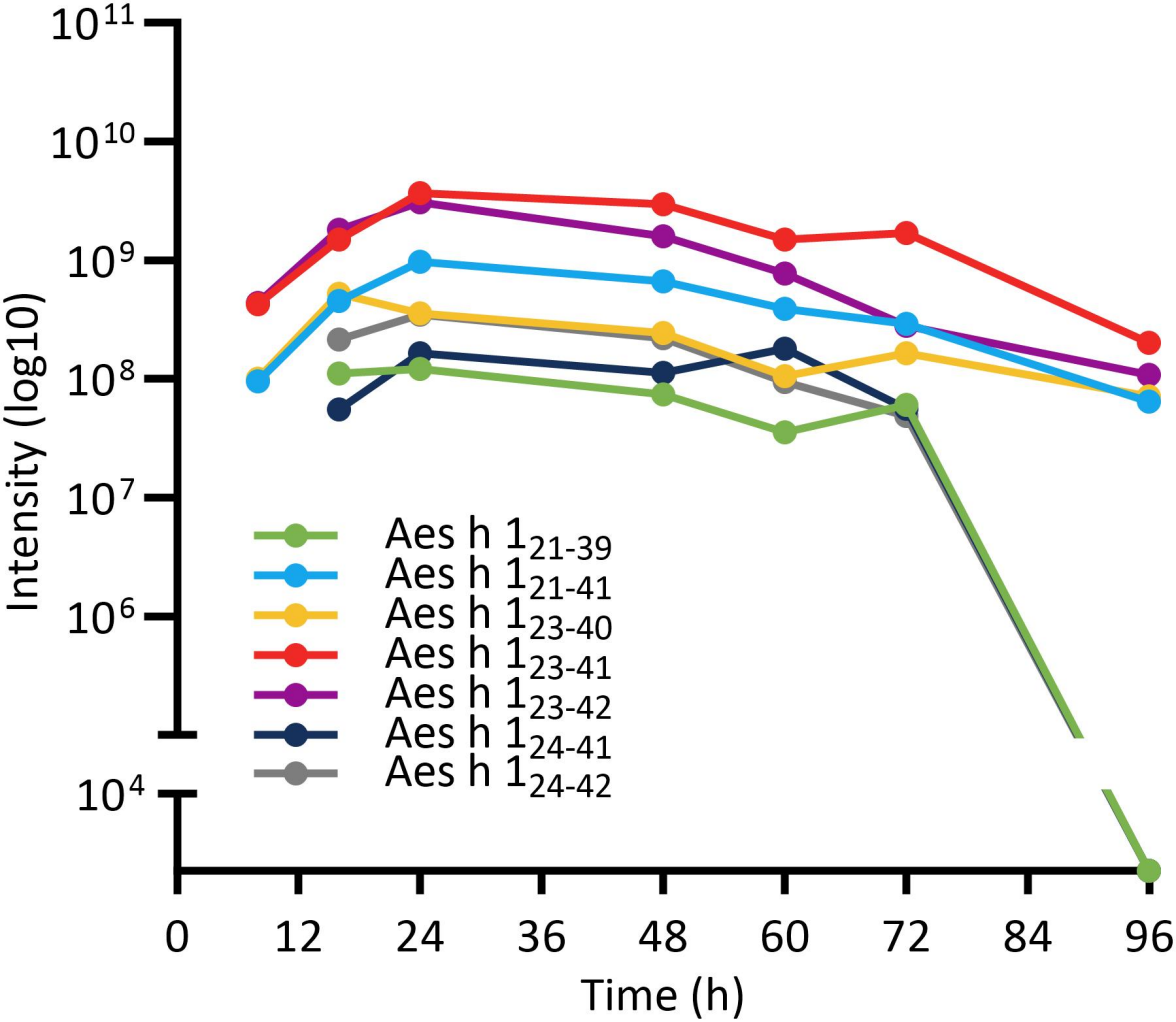
Degradation kinetics of peptides containing Aes h 1_24−35_. Intensity values of peptides containing the Aes h 1_24−35_, which corresponds to the immunodominant T cell epitope Art v 1_25−36_ were monitored for 96 hours. Epitope containing peptides were selected based on the following criteria: **(i)** length between 18 and 21 amino acids; (ii) continuous longitudinal presence with detection at a minimum of 3 time points; and (iii) ± 7 amino acids as flanking N- and C- terminal residues. Peptides with signal intensities below 25% across all time points were excluded.

As Aes h 1 comprises only 51 residues, we explored the option to detect long peptides covering most of the protein. Indeed, we found a multitude of peptides that span up to 92% of the sequence. Aes h 1_5−44_, was present until 72 hours with a consistent intensity until 24 hours (Supplementary Figure S5). The extended presence of this long peptide during endolysosomal degradation confirmed the high protein stability observed in gel electrophoresis.

## 4 Discussion

Endolysosomal processing is a critical step in antigen presentation, therefore characterization of the proteins during the process provides strong insights regarding immunogenicity. In the present work, we developed an optimized protocol for the endolysosomal degradation assay by providing information on pH maintenance, *a priori* cathepsin activity measurement, and addition of a reduction/alkylation step for improved endolysosomal peptide determination using mass spectrometry. Furthermore, the storage stability of endolysosomal proteases was tested and an open-source browser-based application designated PeptideStudio was developed for enhanced visualization of the endolysosomal degradome.

The endolysosomal degradation assay mimics processing of exogenous proteins within the endolysosomal compartments of APCs (1, 2). The stability of proteins during processing in endolysosomes affects the abundance and kinetics of peptide presentation to naïve T cells. The assay was previously used to investigate the immunogenicity of proteins, especially allergens, and to distinguish high and low allergenic variants (2, 4, 7, 8, 14, 24, 25). However, it became evident that results lacked comparability due to high inter assay variability and thus enhanced standardization and reproducibility of the protocol was warranted (1).

The most evident variation across prior studies is the endolysosomal half-life of Bet v 1. It was determined to range from 2 to 16 hours in studies performed with endolysosomal proteins from the JAWSII cell line (2, 6, 18, 26, 27). In addition, different endolysosomal peptide numbers which caused a variation in the apparent dominance of clusters between studies were observed (2, 6). In this context, the pH of the endolysosomal degradation assay was an important feature for providing physiological conditions and assay consistency. This is substantiated by a study using different buffers at pH 4.5, 5.2 and 5.9, which resulted in highly divergent Bet v 1 half-lives of 0.5, 7 and >48 hours, respectively (4). In addition, the relevant antigen processing enzyme cathepsin S was shown to bind Bet v 1 in a pH-dependent manner, with higher binding affinity at pH 4.5 (28). Interestingly, endolysosomal degradation of allergenic tropomyosins revealed a slower degradation and lower peptide amount of Der p 10 and Ani s 3 at pH 4.5 compared to pH 5.2 (14). To ensure comparison between different proteins, maintenance of pH 4.8 in the assay and inclusion of limitations on the buffers of the investigated proteins were integrated into the protocol for optimization of the assay.

The endolysosomal degradation buffer contains 0.1 M citrate buffer to provide physiologically relevant pH 4.8. Due to the polyanionic nature of citrate, high buffer concentrations like 1 M induce strong electrostatic interactions and activity, which influence the pH measurement (29). This phenomenon was highly pronounced and observable during the preparation of the citrate buffer stock. Therefore, we recommend preparing the citrate stock buffer exactly according to our protocol to guarantee correct and comparable assay conditions. Another factor influencing the pH condition is the buffer in which the protein under investigation is dissolved. Proteins in pH 6 buffers reached the desired stable pH of 4.8. However, buffers with a pH ≥7 at high molarity could not preserve the pH 4.8. Thus, recommendations on low concentrated proteins and unfavorable buffer conditions were added to the optimized protocol and protein concentration and/or buffer exchange before use in the endolysosomal degradation assay is suggested. For conditions that fall outside the range tested in this study, verifying the pH of a simulated endolysosomal degradation with all buffer components is highly recommended.

Antigen processing within endolysosomes of APCs is mostly attributed to cysteine cathepsins (30). Inhibition of cysteine cathepsin S during endolysosomal degradation of the major olive pollen allergen Ole e 1 caused a significant delay in degradation. Additionally, the peptide number was reduced causing loss of some prominent peptide clusters (8). Endolysosomal proteins can display varying cathepsin activities due to experimental handling, starting from cell culture to the final isolation of the endolysosomal content. Therefore, we integrated the cathepsin activity measurement into the optimized protocol to adjust the endolysosomal protein amount based on activity. To demonstrate the enhancement in the standardization of the assay, we selected allergens with different endolysosomal degradation profiles. Bet v 1 was used as an example for a susceptible protein while Aes h 1 represents a protein with more resistance to endolysosomal degradation.

Endolysosomal proteins with 2.1-fold higher cathepsin activity resulted in a 1.7-fold decrease in the half-life of Bet v 1 and Aes h 1. Implementation of the endolysosomal protein adjustment based on the endolysosomal constant value of 10 µM overcame this variation. Upon adjustment, comparable half-lives were obtained when using endolysosomal protein batches with different activities. Protocol optimization by adjustment of the endolysosomal protein amount based on cathepsin activity thus provides comparable and internally consistent half-life determinations that will limit batch-to-batch variations of endolysosomal activities.

The endolysosomal half-life calculations were additionally standardized using the one-phase association formula for calculation (21). Previous studies typically presented gel images either without quantitative analyses or reported point-to-point plots in which the half-life was inferred from a putatively linear segment crossing the 50% degradation value (4, 10, 13, 14, 18, 27, 31, 32). This strategy could not appropriately model the underlying degradation kinetics and thus introduced ambiguity in the half-live determination. We thus integrated a curve fitting using a one-phase association model to calculate the half-life using all data points, with the curve constrained to start at 0 and plateau at 100% degradation. The decision of analyzed timepoints in endolysosomal degradation depends on protein stability. Typically, degradation is monitored up to 24 hours for susceptible proteins while highly stable proteins are monitored up to 72 hours (1). To obtain an accurate final curve fit, time points starting from 0.5- or 1-hour degradation should be selected.

Until now, endolysosomal proteins from dendritic cells were consistently isolated one day before endolysosomal degradation. While this procedure suggested highest protease activity, it lacked time efficiency as it required “fresh” endolysosomal purification before each degradation experiment. Thus, we tested storage of endolysosomal proteins at −70 °C and demonstrated stable protease activity for up to 63 days. This knowledge now allows the preparation of larger batches of endolysosomal proteins that can be safely used over a longer period.

In this study, the Aes h 1 endolysosomal degradation profile was investigated for the first time. The horse chestnut allergen presents moderate resistance to endolysosomal degradation indicated by a half-life of 16 hours. Notably, the major mugwort pollen allergen Art v 1 presents a higher resistance to endolysosomal degradation, with the defensin domain determined to be stable up to 72 hours. In comparison, the homologous allergens Amb a 4 and Par h 1 are more susceptible to endolysosomal proteases since their defensin domains were mostly degraded at 8 and 16 hours, respectively (24). These findings indicate that comparative analysis of endolysosomal degradation among allergens within the same protein family is necessary to elucidate their distinct allergenicity profiles.

Peptidomics, qualitative and quantitative analysis of peptides, is an emerging field (33) In this context, the endolysosomal degradomics is an additional versatile application of peptidomics. Available peptide analysis and visualization tools, however, do not adequately meet the requirements of endolysosomal degradome visualization. Peptide-level color coding by intensity and display of amino acid residues rather than abstract lines alone, is central to data interpretation and T cell epitope-focused analyses. Furthermore, effective visualization of endolysosomal degradomes requires inclusion of multiple time points and flexible customization for generation of a figure. Above mentioned features are not readily supported by existing tools, which were designed for different analytical objectives (34–36). Therefore, we developed PeptideStudio to enhance graphical representation and analysis of endolysosomal peptides. This tool integrates semi-quantitative information by mapping peptide intensities directly to individual peptides. It supports color-coded intensity overlays; manual sequence input; selection and display of specific time points; and user-adjustable parameters such as peptide length filters, intensity thresholds, figure and font sizes. Users can also annotate protein regions containing experimentally validated T cell epitopes or modified amino acids. Together, these features strongly enhance the visualization, interpretation and comparison of endolysosomal degradomes.

For proteins stabilized by disulfide bonds, a reduction and alkylation step was integrated after endolysosomal degradation to prevent the formation of artificial inter-peptide crosslinks or intra-peptide loop formation (37). Those peptide assemblies would not be detected in mass spectrometry due to lacking matches with the reference sequences. Implementation of a reduction and alkylation step after endolysosomal degradation preserved the cysteine-containing peptides for correct identification in mass spectrometry, which was successfully demonstrated with the disulfide bond stabilized allergen Aes h 1.

Our work corroborates previous studies on Bet v 1 confirming that T cell epitopes are localized within prominent peptide clusters (6, 7, 38). Overall, Bet v 1 displayed more peptides which can be attributed to enhanced sensitivity in mass spectrometry measurements. Degradation kinetics of endolysosomal peptides containing experimentally validated Bet v 1 T cell epitopes appeared early and persisted for a moderate period. This supports a prior hypothesis that early and sustained peptide persistence can enhance MHC II loading and antigen presentation, consistent with the role of Bet v 1 as a primary sensitizer and major allergen (7, 39).

The endolysosomal degradome of Aes h 1 revealed two prominent clusters at the N- and C-terminus similar to other allergenic defensins (24). Aes h 1 showed a delayed peptide profile kinetics, with peptide counts and total intensities peaking at 24 hours. This kinetic corresponds to the experimentally observed half-lives, supporting the idea that epitope generation is shaped by intrinsic stability and structural features of proteins during endolysosomal processing. Notably, peptides including Aes h 1_24−35_ which correspond to the immunodominant T cell epitope of Art v 1_25−26_ (67% sequence identity) (40), were identified with high intensities.

By leveraging endolysosomal degradome profiling, we provide an orthogonal line of evidence for half-life measurements and extend prior observations on endolysosomal stability. This approach highlights how endolysosomal degradation can modulate the temporal window of T cell epitope availability, with potential implications for antigen presentation and T cell priming. Together, these findings underscore that degradome kinetics can serve as an informative proxy for antigen processing behavior and help explain differences in immunogenicity between allergens with distinct structural properties.

A study limitation includes the use of endolysosomal proteases from a murine cell line. While similar kinetics and degradation profiles using murine and human dendritic cells were previously shown (2), minor differences in specific proteases or activities cannot be fully ruled out. In terms of standardization, a cell line provides more uniformity than primary cells. However, future applications might also investigate usage of other (human) antigen presenting cells. In mass spectrometry, identification of peptides is subject to intrinsic peptide features and their ionization capacity. Thus, some peptides might be underrepresented, and it should also be noted that intensity values are considered semi-quantitative measures. In previous publications, endolysosomal degradation and mass spectrometry analysis were conducted as single measurement per timepoint. While biological and/or technical replicates may produce more robust statistical outcomes, it would also lead to a substantial increase in the number of mass spectrometry runs.

The optimization of the endolysosomal degradation assay was achieved by providing precise protocols for the maintenance of the pH conditions and the incorporation of the constant endolysosomal activity value. This ensures consistency in cathepsin activity across different endolysosomal protein preparations and thus comparability for degradation kinetics and half-life determination. For the first time, endolysosomal peptides were alkylated to ensure that peptides from disulfide-bond containing proteins are efficiently detected in mass spectrometry. We further developed PeptideStudio which includes several customizable features for effective representation of peptides obtained from endolysosomal degradation.

In conclusion, the optimized and standardized protocol developed in this study improves reproducibility and comparability across studies. Systematic investigation of allergens from high- and low-allergenic sources within the same protein families will strengthen our knowledge regarding endolysosomal degradation profiles and *in silico* predictions. Overall, the standardized assay represents a robust, orthogonal method that can be used to predict protein immunogenicity and allergenicity, thereby contributing to the safety assessment and risk evaluation of novel foods or other potentially immunogenic proteins.

## Data Availability

The original contributions presented in the study are included in the article/Supplementary Material, further inquiries can be directed to the corresponding author.
