# Supplementary material for: The optimized endolysosomal degradation assay with standardized enzyme activity represents an orthogonal method for predicting protein immunogenicity and T cell epitopes
Source: Front Allergy. 2026 Sep 23;7:1936335. doi: 10.3389/falgy.2026.1936335 (PMC13643861; doi:10.3389/falgy.2026.1936335)
Supplement: Supplementary file 1 [file Datasheet1.pdf]

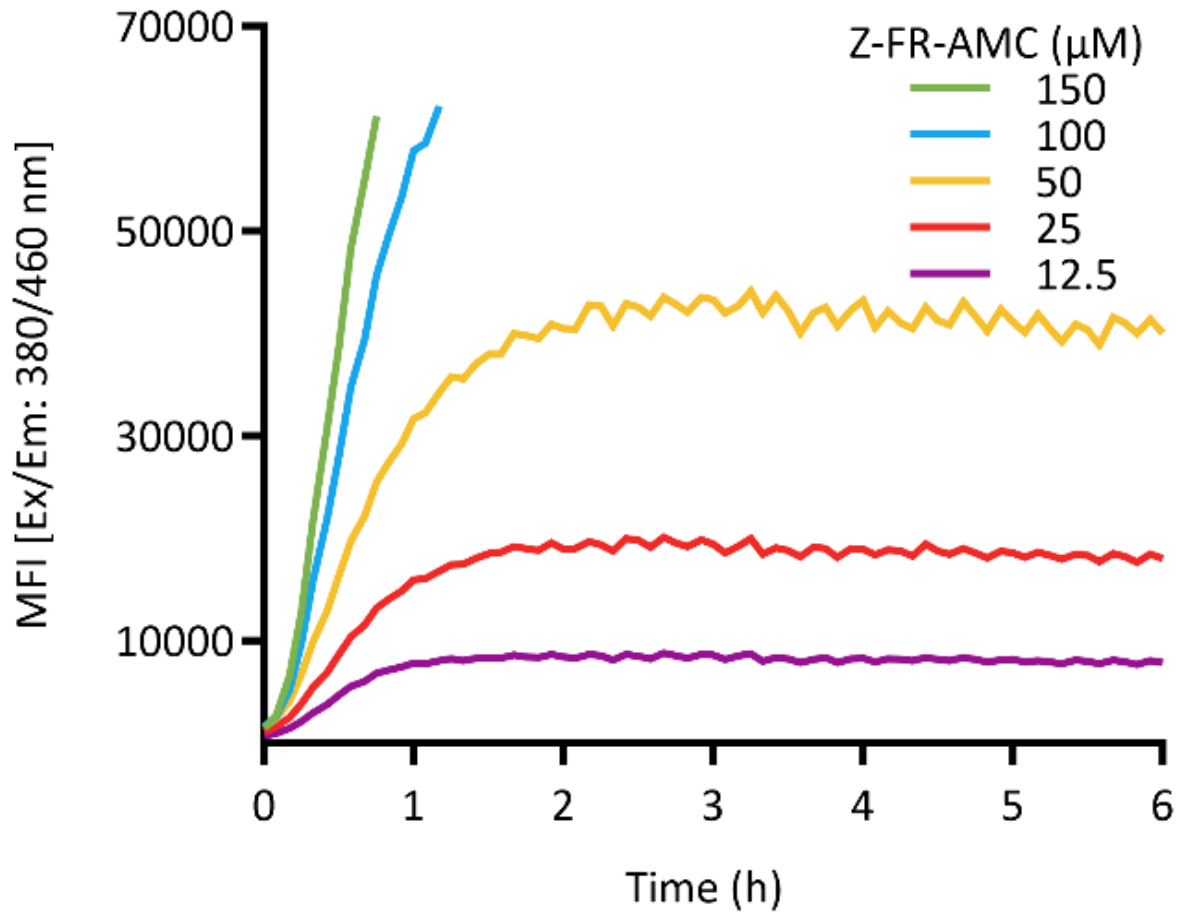

Supplementary Figure 1. Z-FR-AMC concentration optimization for the cathepsin activity assay. To optimize the cathepsin activity measurement of endolysosomal proteins, different substrate concentrations of Z-FR-AMC ranging from 12.5 to 150 µM were tested with 2.5 µg endolysosomal proteins.
