# Supplementary material for: The optimized endolysosomal degradation assay with standardized enzyme activity represents an orthogonal method for predicting protein immunogenicity and T cell epitopes
Source: Front Allergy. 2026 Sep 23;7:1936335. doi: 10.3389/falgy.2026.1936335 (PMC13643861; doi:10.3389/falgy.2026.1936335)
Supplement: Supplementary file 2 [file Datasheet2.pdf]

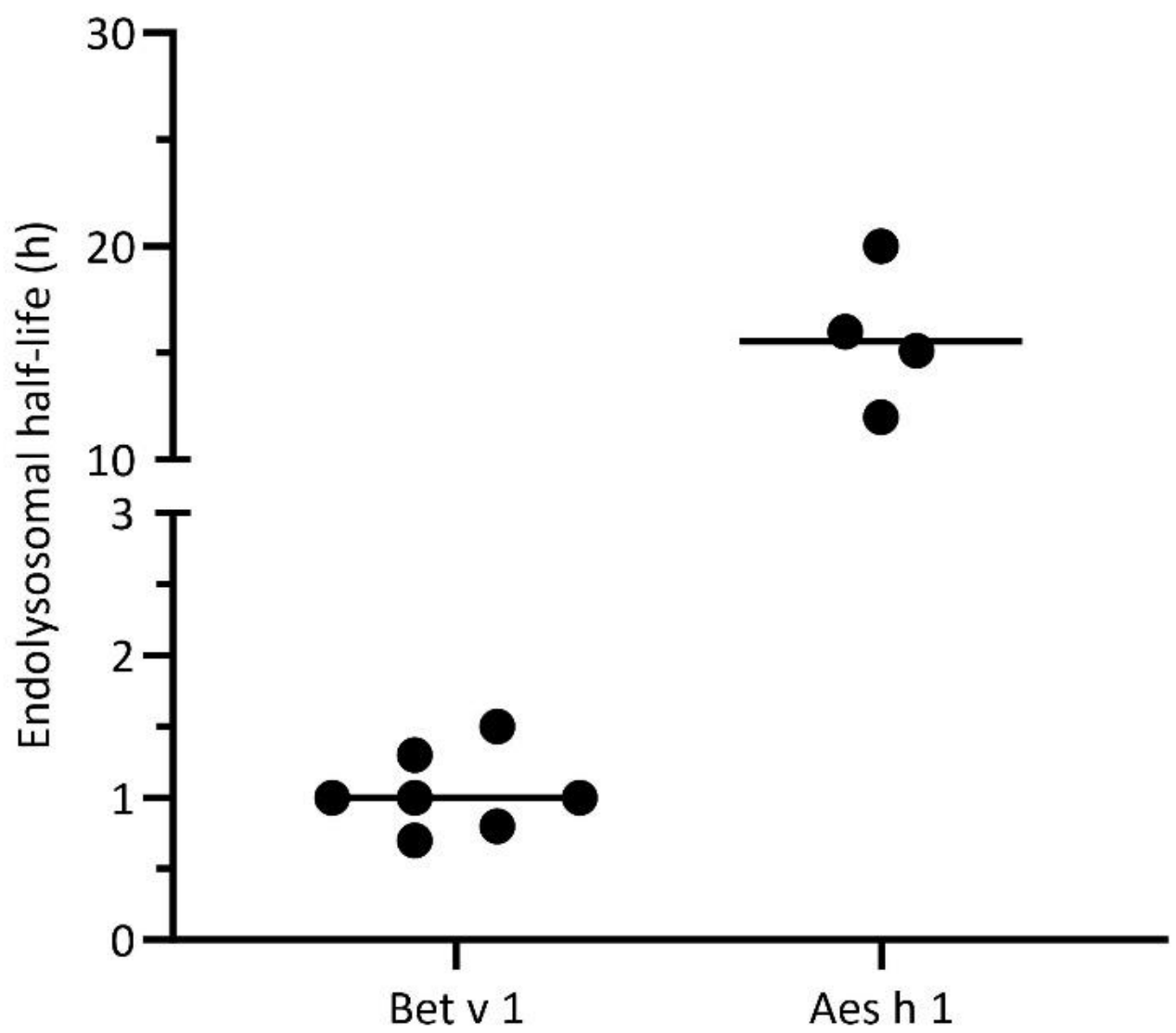

	Bet v 1	Aes h 1
Number of values	7	4
Mean	1	16
Std. Deviation	0.28	3.3
Std. Error of Mean	0.1	1.6

Supplementary Figure 2. Endolysosomal half-lives of Bet v 1 and Aes h 1. Endolysosomal half-lives of Bet v 1 and Aes h 1 determined using independent endolysosomal protein batches (Bet v 1: n = 7; Aes h 1: n = 4). Number of values, mean, standard deviation, and standard error of the mean are reported in the table.
