# Supplementary material for: The optimized endolysosomal degradation assay with standardized enzyme activity represents an orthogonal method for predicting protein immunogenicity and T cell epitopes
Source: Front Allergy. 2026 Sep 23;7:1936335. doi: 10.3389/falgy.2026.1936335 (PMC13643861; doi:10.3389/falgy.2026.1936335)
Supplement: Supplementary file 3 [file Datasheet3.pdf]

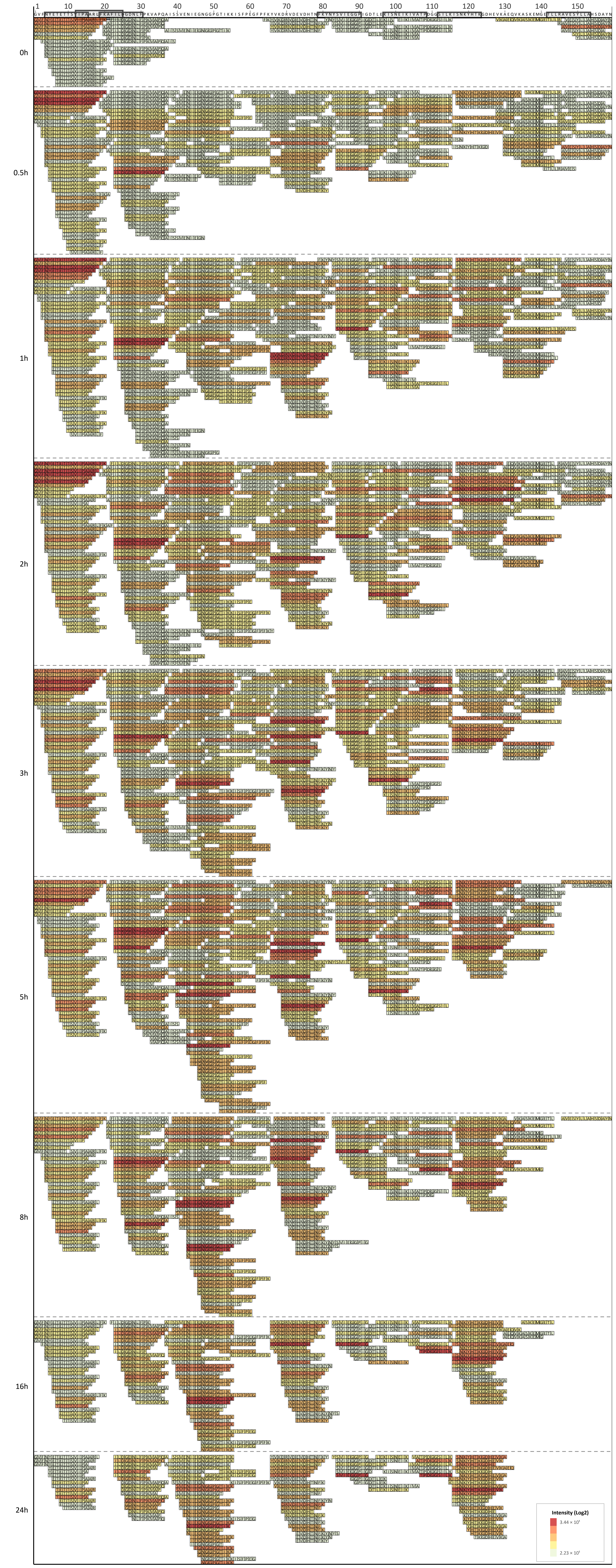
1 10 20 30 40 50 60 70 80 90 100 110 120 130 140 150
GVFNYETETTSVIPAARLFKAFILDGDNLFPKVAPQAISSVENIEGNGGPGTIKKISFPEGFPFKYVKDRVDEVDHTNFKYNYSVIEGGPIGDTLEKISNEIKIVATPDGGSILKISNKYHTKGDHEVKAEQVKASKEMGETLLRAVESYLLAHSDAYN
0h
0.5h
1h
2h
3h
5h
8h
16h
24h
Intensity (Log2)
3.44 × 10¹
2.23 × 10¹
