# Supplementary material for: The optimized endolysosomal degradation assay with standardized enzyme activity represents an orthogonal method for predicting protein immunogenicity and T cell epitopes
Source: Front Allergy. 2026 Sep 23;7:1936335. doi: 10.3389/falgy.2026.1936335 (PMC13643861; doi:10.3389/falgy.2026.1936335)
Supplement: Supplementary file 4 [file Datasheet4.pdf]

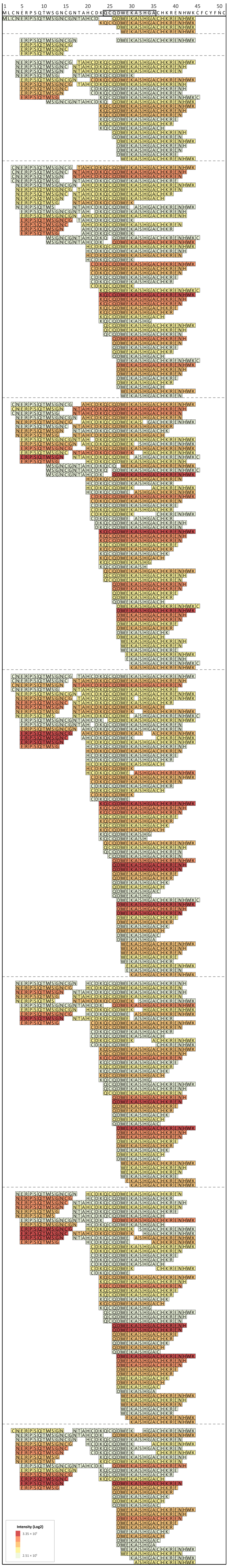

1 5 10 15 20 25 30 35 40 45 50
MLCNERPSQTWSGNCGNTAHCDKQCQDWEKASHGACHKRENHWKCFCYFNC
0h
3h
8h
16h
24h
48h
60h
72h
96h
Intensity (Log2)
