# Supplementary material for: The optimized endolysosomal degradation assay with standardized enzyme activity represents an orthogonal method for predicting protein immunogenicity and T cell epitopes
Source: Front Allergy. 2026 Sep 23;7:1936335. doi: 10.3389/falgy.2026.1936335 (PMC13643861; doi:10.3389/falgy.2026.1936335)
Supplement: Supplementary file 5 [file Datasheet5.pdf]

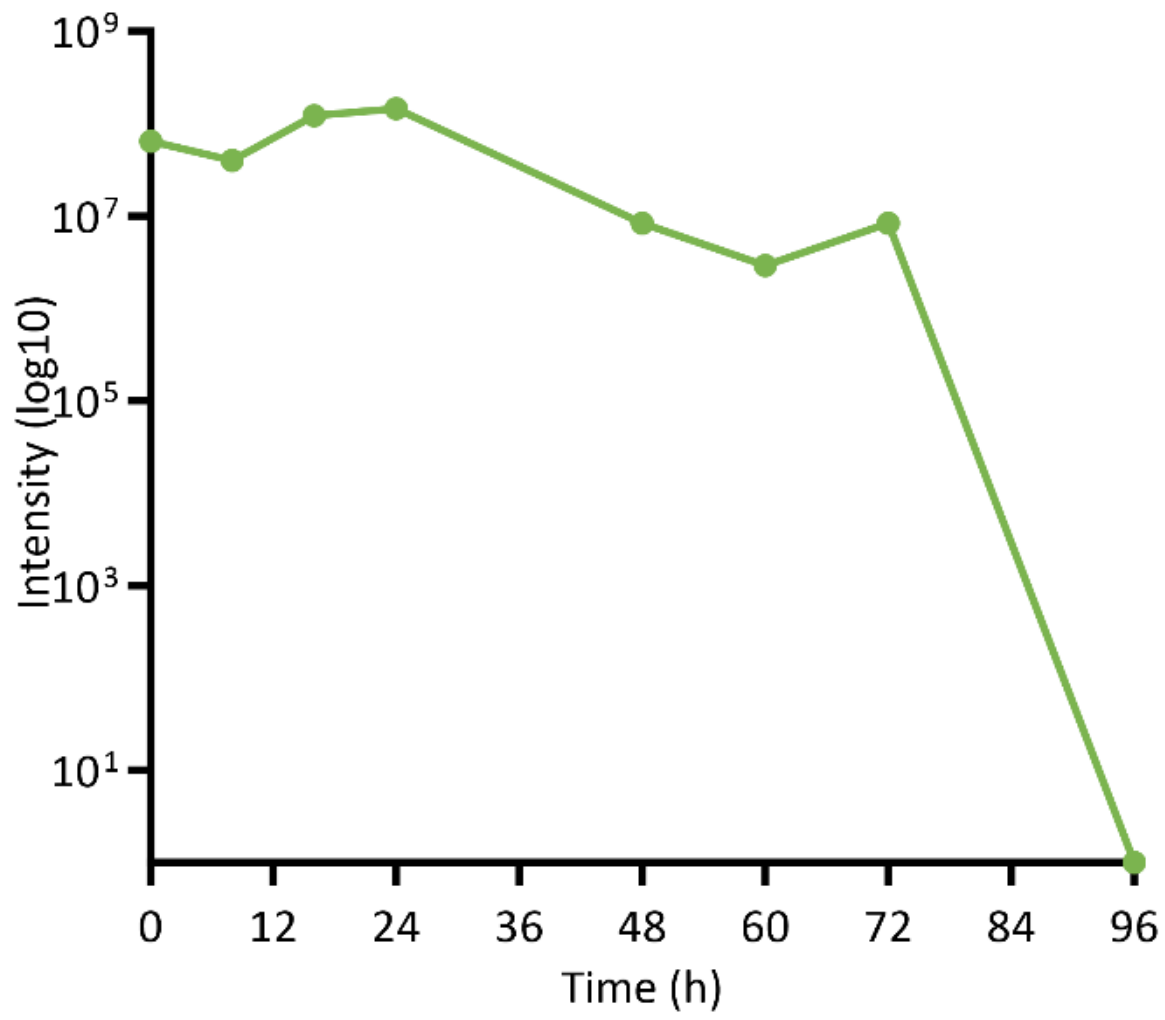

Supplementary Figure 5. Degradation kinetics of Aes h $1_{5\text{-}44}$. Intensity values of Aes h $1_{5\text{-}44}$ peptide were monitored for 96 hours. Aes h $1_{5\text{-}44}$ spans 78% of the full-length Aes h 1 sequence.
